# Quantifying satisfaction among pharmacists working in Pharmaceutical Sales or Marketing and its inferential relationship with demographics: A Cross-Sectional analysis in Pakistan

**DOI:** 10.12669/pjms.37.2.3447

**Published:** 2021

**Authors:** Muhammad Hammad Butt, Shahzadi Misbah, Abrar Ahmad, Tooba Mehboob, Irfan Bashir

**Affiliations:** 1 Muhammad Hammad Butt, Faculty of Pharmacy, University of Central Punjab, Lahore, Pakistan; 2 Shahzadi Misbah, Faculty of Pharmacy, University of Central Punjab, Lahore, Pakistan; 3 Abrar Ahmad, Faculty of Pharmacy, University of Central Punjab, Lahore, Pakistan; 4 Tooba Mehboob, Faculty of Pharmacy, University of Central Punjab, Lahore, Pakistan; 5 Irfan Bashir Foundation for Young Researchers, Lahore, Pakistan, Faculty of Pharmacy, University of Central Punjab, Lahore, Pakistan

**Keywords:** Factors, Job satisfaction, Medical sales representative, Pharmaceutical marketing, Pharmacist

## Abstract

**Objectives:**

To quantify satisfaction among pharmacists working in pharmaceutical sales or marketing in Pakistan.

**Methods:**

A cross-sectional study was conducted among pharmacists working in pharmaceutical sales and marketing during August to November, 2019. Satisfaction score of pharmacists was considered satisfied (Score > 2.5) and dissatisfied (Score < 2.5). The maximum and minimum satisfaction score was four and one for each question respectively.

**Results:**

A total of 250 questionnaires were distributed and 200 were received back yielding a response rate of 80%. Male respondents dominated the cohort and were more satisfied (2.60 ± 0.47) as compared to females (2.31 ± 0.48) with majority were 18-30 years old and 78% had Pharm. D level of education (2.61 ± 0.47). The mean satisfaction score of participant is 2.51 with standard deviation of 0.49. The 53% of the respondents were dissatisfied with their salaries. The study dependent and independent variables are correlated with each other and significant results were seen between them. The factors associated with dissatisfaction are fear of losing job (2.19 ± 0.88), switch job (2.20 ± 0.77) and health condition (2.09 ± 0.89). Factors associated with satisfaction are positive attitude of doctor (2.91 ± 0.60), doctor consider respectful job holder (2.91 ± 0.61), enjoy work (3.01 ± 0.65), job by choice (3.12 ± 0.67) and progress in job (3.00 ± 0.74).

**Conclusions:**

It was concluded that the respondents were dissatisfied due to number of issues including disturbance in their personal life, lack of promotion and incentives among study participants.

## INTRODUCTION

Job satisfaction is a measure of employees’ contentedness with their job and its various facets or aspects.^1^ Numerous studies show remarkable impact of employees’ job satisfaction on their motivation, and the level of motivation impacts performance and productivity in an organization.^2^ Several jobs associated factors including salary, undefined promotion criteria and lack of encouragement may lead to depressive symptoms among employees. Satisfaction enhances with prize & pay level in term of company expenditure returns.^3^

Pharmaceutical industry in Pakistan essentially depends on the doctors’ prescriptions consisting of their products’ brand names. For this reason, the industry has largely adopted the strategy of personnel selling in which Medical Representatives from the pharmaceutical companies meet clinical practitioners, make sales call and tries to convince and motivate them to write their brands in their prescriptions by sharing scientific information and competitive advantages over their competitions. The job position of medical representative has historically been occupied by non-medical or non-scientific personnel in Pakistan usually having only a bachelor in arts degrees. Keeping in view the comprehensive knowledge in pharmaceutical sales and marketing (S&M), Pharmacists are the ideal candidates for these positions. However, very less number of pharmacists opt this job after graduation in Pakistan. Moreover, turnout of pharmacists from pharmaceutical S&M is much higher than any other pharmacy profession. This high turnover might be attributed to poor job satisfaction, tedious nature of job, unhealthy attitude of other healthcare professionals towards pharmaceutical sales representatives, pressure to achieve the targets, and threat of losing the job.^4^,^5^ Since pharmacists are responsible for provision of healthcare related information, increased satisfaction among pharmacists may result in good healthcare services and better productivity.^6^

Very few studies have been performed to find the relationship between job satisfaction among pharmacists with their performance,^7^ where low satisfaction could explicitly translate into low performance.^8^ However, very few studies explored satisfaction of pharmacists in marketing and sales department. In this context, it is of utmost importance to explore the satisfaction levels and motivating factors among pharmacists working in pharmaceutical S&M. There are number of methods available to ascertain the job satisfaction and its impact on physical and mental health.^9^ To the best of our knowledge, there is no study in Pakistan evaluating the job satisfaction among pharmacists in pharmaceutical S&M. This study is perhaps first of its kind which explores the satisfaction level among pharmacists working as S&M representatives and underscores their job-related problems.

## METHODS

The cross-sectional survey was conducted among pharmacists working as S&M representatives in different pharmaceutical organizations in Pakistan form August-November, 2019. The study was approved by Institutional Ethics Committee (IEC) of University of Central Punjab (Ref: # UCP\FOP\315) and from Research and Ethics Committee (REC) of Foundation for Young Researchers, Lahore, Pakistan (Ref. # FYR/R&EC/09/2019). Before completing the questionnaire, informed consent was taken from the participants. The sample size for the current study was 185 calculated by Raosoft®, with 80% test power, 5% error margin, 90% confidence interval and 50% response distribution.

The 25-items questionnaire was developed by in depth literature research having close ended questions in section one of demographics and section two of job satisfaction and associated factors. The survey instrument was pretested (n = 30), with the purpose of evaluating the clarity and face validation of questions. The reliability scale was applied for these 30 respondents and the alpha value was found at 0.721, indicating the adequacy of the tool to meet the objectives of the current study. The four options likert scale was used to assess the satisfaction level of pharmacists, and factor associated with job. Satisfaction scoring criteria were used as ‘Strongly Agree’ indicated with a score of four, ‘Agree’ indicated with a score of three, ‘Disagree’ indicated with a score of two and ‘Strongly disagree’ indicated with a score of one for positive sense questions (Q1-Q9) and for negative sense questions (Q10-Q18) satisfaction score were reversed. The maximum and minimum satisfaction score was four and one for each question respectively. Satisfaction score (SS) of pharmacist was considered satisfied (score > 2.5) and dissatisfied (score < 2.5). The scoring system used in this study has been previously used in several published studies.^10^,^11^

**Fig.1:**
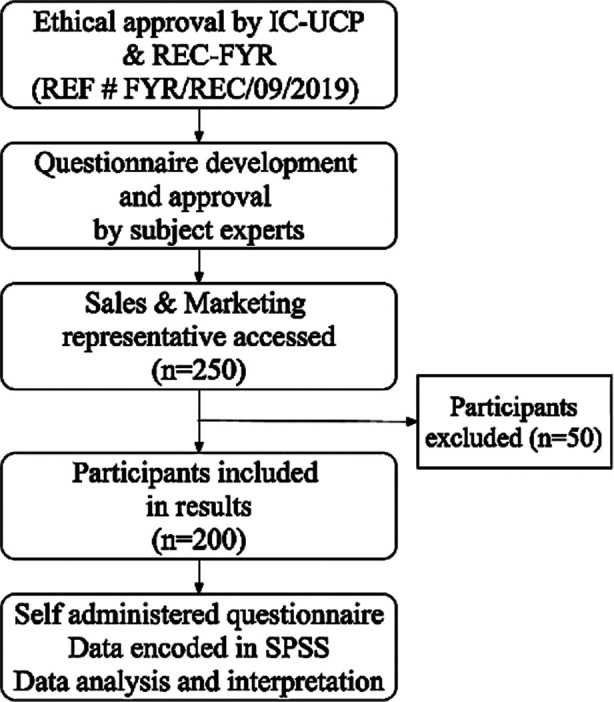
Study Flow Diagram.

Before filling the questioner, the objective of the study was clarified to each participant and participants who agreed were requested to fill the questionnaire. The data was analyzed using Statistical Package for Social Science (SPSS) version 21.0.0. All categorical variables were described using frequency (N) and percentages (%). The relationship between dependent and independent variables was evaluated by using correlation coefficients. A p-values <0.05 were considered statistically significant. The study flow diagram is presented in Fig.1.

## RESULTS

Out of total 250 questionnaires distributed,, 200 were returned yielding a response rate of 80%. Males dominated the cohort 70.5%. Majority of the respondents (85.5%) belonged to the age group of 18-30 years with mean SS of 2.51 ± 0.50. Forty respondents had Master (M.Phil.) level of education with mean SS of 2.15 ± 0.42 while 78% respondents had Pharm D with mean SS of 2.61±0.47. The complete demographics of the study respondents is shown in Table-I. Among all respondents the cumulative mean SS comes out to be 2.51 with S.D of 0.49. All of the respondents were directly responsible for the S&M job. The responses of attitude, satisfaction and factor associated questions with their Score (Mean and Standard Deviation (S.D)) are presented in Table-II.

**Table-I:** Socio-Demographic Profile.

*Attributes*	*Frequency*	*Percentage*	*Score*

	*(N)*	*(%)*	*Mean ± S.D*
***Age (Years)***			
18-30	171	85.5	2.51 ± 0.50
31-45	27	13.5	2.51 ± 0.47
45+	2	1	2.67 ± 0.39
***Gender***			
Male	141	70.5	2.60 ± 0.47
Female	59	29.5	2.31 ± 0.48
***Qualification***			
M.Phil	40	20	2.15 ± 0.42
Pharm D	156	78	2.61 ± 0.47
B Pharmacy	4	2	2.31 ± 0.35
***Experience (Years)***			
< 5	146	73	2.52 ± 0.54
5-10	42	21	2.41 ± 0.29
11-15	5	2.5	2.98 ± 0.47
> 15	7	3.5	2.67 ± 0.32
***Salary (Pakistani Rupees)***			
< 40,000	80	40	2.60 ± 0.49
40000-55000	75	37.5	2.32 ± 0.50
55000-65000	20	10	2.60 ± 0.37
> 65000	25	12.5	2.75 ± 0.39
***Work Place***			
Multinational	120	60	2.36 ± 0.42
Local	57	28.5	2.61 ± 0.52
Others (franchisers, local trading)	23	11.5	3.06 ± 0.36
***Duty hours Per day***			
< 8 Hours	39	19.5	2.91 ± 0.53
8-10 Hours	143	71.5	2.40 ± 0.43
11-12 Hours	11	5.5	2.54 ± 0.56
> 12 Hours	7	3.5	2.52 ± 0.49

**Table-II:** Participant’s attitude with their responses and score.

*Study variables*	*Strongly agree*	*Agree*	*Disagree*	*Strongly disagreed*	*Score*

	*N (%)*	*N (%)*	*N (%)*	*N (%)*	*Mean ± S.D*
Doctor give you a positive attitude	27 (13.5%)	128 (64%)	44 (22%)	1 (0.5%)	2.91 ± 0.60
Satisfied with your job after Pharmacy degree	40 (20%)	75 (37.5%)	83 (41.5%)	2 (1%)	2.77 ± 0.78
Satisfied with your salary	44 (22%)	50 (25%)	102 (51%)	4 (2%)	2.67 ± 0.84
Enjoy your work	43 (21.5%)	115 (57.5%)	42 (21%)	None	3.01 ± 0.65
See any progress with sales & marketing job	54 (27%)	93 (46.5%)	52 (26%)	1 (0.5%)	3.00 ± 0.74
Doctor consider you a respectful job holder	29 (14.5%)	123 (61.5%)	48 (24%)	None	2.91 ± 0.61
Doing the job with your choice	56 (28%)	113 (56.5%)	29 (14.5%)	2 (1%)	3.12 ± 0.67
Doctor think you are an educated person and pay attention towards discussion	69 (34.5%)	83 (41.5%)	45 (22.5%)	3 (1.5%)	3.09 ± 0.79
Satisfy with your work and life balance	25 (12.5%)	95 (47.5%)	71 (35.5%)	9 (4.5%)	2.68 ± 0.75
Have the fear of losing the job	48 (24%)	80 (40%)	58 (29%)	14 (7%)	2.19 ± 0.88
You often think to switch your job	41 (20.5%)	81 (40.5%)	76 (38%)	2 (1%)	2.20 ± 0.77
Job affect your health like depression, anxiety, High blood pressure	59 (29.5%)	77 (38.5%)	52 (26%)	12 (6%)	2.09 ± 0.89
Your job is challenging	85 (42.5%)	78 (39%)	36 (18%)	1 (0.5%)	1.77 ± 0.76
Pay is less than your work	63 (31.5%)	80 (40%)	56 (2%)	1 (0.5%)	1.98 ± 0.79
Incentive is less than your performance	51 (25.5%)	80 (40%)	68 (34%)	1 (0.5%)	2.10 ± 0.78
Criticism if you didn’t complete your targets provided	42 (21%)	92 (46%)	63 (34%)	3 (1.5%)	2.14 ± 0.75
Wish to switch your job due to depression, anxiety, High Blood pressure	15(7.5%)	67(33.5%)	111(55.5%)	7(3.5%)	2.55 ± 0.69
Job influenced your personal life and family time	43 (21.5%)	97 (48.5%)	52 (26%)	8 (4%)	2.13 ± 0.79

The determined correlation between dependent (salary) and independent (Experience, Age, Duty hours and stress) variables were assessed, as the relation between salary and experience having positive correlation 0.533, means with the experience, the salary was increasing. In the case of age and salary, the observed correlation was 0.497, means both parameters have a positive correlation. As in our study, most of the respondents were at the beginning of their careers at age group 18-30, and this was an important factor which is associated with the satisfaction of the job. While the correlation between salary and duty hours were slightly less positive at 0.438. This was not a strong factor which associates with the job satisfaction. As in every case, increasing duty hours result in increased output in terms of salary. While stress and salary were having a negative correlation -0.057, which yields that increase in salary causes a decrease in stress.

## DISCUSSION

To the best of our knowledge, this study is perhaps the first study to explore estimation of pharmacists’ satisfaction and its correlation with job related factors in Pakistan. A previous relevant study conducted in Pakistan^3^ did not included pharmacist working as S&M representative. However, we limited our analysis to pharmacists in the S&M department. The overall objective of this study was to evaluate the satisfaction level of a pharmacist in S&M jobs and determine risk factors associated with dissatisfaction towards the job. Many factors contribute towards job satisfaction which is sometimes difficult to control,^12^ such as culture within the organization can drastically influence satisfaction and performance of employees.^13^ Both individual and organizational factors contribute satisfaction of job.^14^

Job satisfaction is mainly influenced by self-confidence and doctors’ attitude. However, more than half of the participants were satisfied with their jobs and reported to be well treated during meeting with the doctors, majority were satisfied with the positive attitude (Mean SS 2.91± 0.60) and consider them a respectful job holder (Mean SS 2.91 ± 0.61). The factors associated with satisfaction in the current study corroborate with those observed by another study conducted in a corporate organization in Pakistan.^15^ The results of this study showed good satisfaction and demonstrated good support from doctors, heads and colleagues in improving work-life balance. These findings are consistent with another study conducted in Pakistan demonstrating the satisfaction among medical representative using similar parameters.^16^ The study highlighted that most of the participants were satisfied with their salaries (Mean SS 2.67 ± 0.84) but showed dissatisfaction when they were asked “pay was less than the work” (Mean SS 1.98 ± 0.79). Similar findings have been reported by another study conducted in Japan where salary was the significant factor associated with dissatisfaction.^17^ It’s the global dilemma that physicians most often consider pharmacist as an inferior health care professional with no medical knowledge or experience and they don’t give due respect to the pharmacists as prestigious healthcare professionals. This was an important factor of concern in job satisfaction of pharmacists in S&M department. In our study, pharmacists were satisfied with the job experience by the respect that was given to them by the physicians. These findings are in contrast to a study, where physicians don’t cooperate with the pharmacists as they were not having appropriate medical knowledge.^18^ This disparity in results might be attributed to the differences in study design or data collection pattern. Job stress was the key factor in job dissatisfaction.

In growing communities, everyone has a lot of ambitions to accomplish by working more to earn a handsome salary. However, majority of the participants (64%) in our study agreed or strongly agreed (Mean SS 2.19 ± 0.88) to having feared losing their job, 61% were agree or strongly agree (Mean SS 2.20 ± 0.77) to thinking of switching the job while 59% (Mean SS 2.55 ± 0.69) actually wished to switch. The reason behind these could be linked with factors such as effects of job on health, as 68% participants agreed or strongly agreed (Mean SS 2.09 ± 0.89) that their job had adverse effects on their health such as depression, anxiety, hypertension etc. Other factors might include less pay compared to their work and less incentives as 71.5% agreed or strongly agreed (Mean SS 1.98 ± 0.79) that they were paid less than their work and 65.5% agreed or strongly agreed (Mean SS 2.10 ± 0.78) that incentives are less than their performance. Additionally, 67% participants also agreed or strongly agreed (Mean SS 2.14 ± 0.75) that they were criticized when they couldn’t meet targets but showed good satisfaction and 70% agreed or strongly agreed (2.13 ± 0.79) that their personal life and family time was adversely affected due to the job.

Pharmacists are core component of any pharmaceutical organization to expand their business from production to sales therefore their satisfaction is of paramount importance in the success of company. Pharmacists have basics of S&M as they are well trained through the course they learnt during the degree program. Provision of opportunities to pharmacists in the S&M department will not only ensure the appropriate and ethical selling and marketing but will also lead to considerable growth in organization’s revenue. Pharmaceutical organizations will need to spend less money on the training of pharmacist due to pre-requisite knowledge they learn during pharmacy program. In order to ensure longer retention of pharmacists, organization should periodically assess their mental health and satisfaction. These findings corroborate with the results of another study reporting the physical and psychological factors associated with job dissatisfaction among pharmacists.^17^ However, the present study is strengthened by the first assessment conducted in whole Pakistan and evaluates the extent of satisfaction in relationship with demographic profile.

### Limitations of the study

This is a cross sectional analysis in Pakistan so more data can be collected from the study population and a nationwide study with greater sample size is need of the hour to quantify the satisfaction level of pharmacist.

## CONCLUSIONS

The medical sales representatives are disturbed with numerous factors which affect their personal life and physical health. Lack of promotion, low salary and incentives were observed as the leading cause of dissatisfaction and physical health which provide hindrance to pharmacist to accomplish their routine tasks. However, overall pharmacist are satisfied with this job. Periodic evaluation of pharmacist`s satisfaction towards job and aggressive maneuvers to address any stressor will not only increase the employee retention time but will also help the organization to achieve its goals in given timeframe.

### Authors` Contribution:

**MHB, AA & IB:** Conceptualized the study.

**MHB, SM & TM:** Did literature search and drafted the study tool.

**MHB, AA & IB:** Analyzed the data and drafted the manuscript.

**SM, AA and TM:** Assisted in interpreting the results.

All authors have approved the final version of manuscript and are responsible and accountable for the accuracy or integrity of the work.
